# Molecular diversity of hepatitis B virus among pregnant women in Amhara National Regional State, Ethiopia

**DOI:** 10.1371/journal.pone.0276687

**Published:** 2022-11-15

**Authors:** Mulat Dagnew, Feleke Moges, Moges Tiruneh, Yihenew Million, Aschalew Gelaw, Mulat Adefris, Yeshambel Belyhun, Uwe G. Liebert, Melanie Maier

**Affiliations:** 1 Department of Medical Microbiology, School of Biomedical and Laboratory Sciences, College of Medicine and Health Sciences, University of Gondar, Gondar, Ethiopia; 2 Department of Virology, Institute of Medical Microbiology and Virology, Leipzig University, Leipzig, Germany; 3 Department of Gynecology and Obstetrics, School of Medicine, College of Medicine and Health Sciences, University of Gondar, Gondar, Ethiopia; Bangladesh Agricultural University, BANGLADESH

## Abstract

**Background:**

Despite the availability of effective vaccines and treatments for hepatitis B virus (HBV), it continues to be a major public health problem in sub-Saharan Africa including Ethiopia. Routine screening for HBV in pregnant women is widely recommended, but there is lack of screening for HBV during pregnancy in Ethiopia. Therefore, this study aimed to assess viral load, and genetic diversity among pregnant women in the Amhara National Regional State, Ethiopia.

**Materials and methods:**

Hepatitis B surface antigen (HBsAg) testing was performed on 1846 pregnant women, 85 of who tested positive were included in this study. HBV DNA was isolated from 85 positive sera, and the partial surface/polymerase gene was amplified and sequenced. HBV genotypes, sub-genotypes, serotypes and mutations in surface genes and polymerase were studied.

**Results:**

Out of 85 pregnant women`s HBsAg positive sera, 59(69.4%) had detectable viral DNA. The median viral load was 3.4 log IU/ml ranging from 2.6 to7.6 and 46 samples were successfully sequenced and genotyped. Genotypes A and D were identified in 39 (84.8%) and 7 (15.2%); respectively. All genotype A isolates were further classified into sub-genotype A1 and serotype adw2 (84.8%) whereas genotype D isolates were further classified into three sub genotypes; 2 (4.3%) D2, 1(2.2%) D4, and 4 (8.7%) D10 with serotypes ayw2 (10.9%), and ayw3 (4.3%). There were 19 (41.3%) surface gene mutations in the major hydrophilic region (MHR). Six (13.1%) of them were discovered in MHR`s `a’-determinant region. Six polymerase gene mutations (13%) were identified.

**Conclusion:**

Genotype A was the predominant genotype in the Amhara National Regional State. The surface and polymerase gene mutations identified in this study may lead to immune therapy failure, diagnostics escape and drug resistance. Thus, the data generated in this study will contribute to the planning of HBV diagnosis, vaccination and treatment, and most importantly to the prevention of vertical transmission of HBV in Ethiopia. Therefore, further molecular studies on HBV are warranted and continuous surveillance is important for patient management and for the prevention and control of HBV infection in the country.

## Background

Viral hepatitis is a major public health problem globally comparable to other major infectious diseases such as human immunodeficiency virus (HIV), tuberculosis and malaria. It is currently the seventh leading cause of mortality globally [1]. Hepatitis B virus (HBV) infects two billion people worldwide with 360 million are being chronically infected. Sixty millions of these chronically infected individuals reside in sub-Saharan Africa [2]. In sub-Saharan Africa, HBV is endemic and the prevalence of hepatitis B surface antigen (HBsAg) is greater than 8% in the general population [3]. Hepatitis B virus infection is responsible for 80% of hepatocellular carcinoma (HCC) cases observed in sub-Saharan Africa. An estimated 90% of HBV chronically infected patients around the world are unaware of their infection. It is a silent killer disease and it is very difficult to identify infection unless properly diagnosed [3]. This number is even higher in sub-Saharan Africa where diagnostic kits are not readily available. Ethiopia is a sub-Saharan African country with the highest HBV infection rate. The seroprevalence of HBsAg was reported as 8.3% in pregnant women and up to 14% in the general population in Ethiopia [4–6].

Mother-to-child transmission (MTCT) at birth is the most common form of HBV transmission in highly endemic areas [7]. Without any intervention, the likelihood of MTCT for a pregnant woman positive for HBsAg, Hepatitis B e antigen (HBeAg), and HBV viral load of deoxyribonucleic acid (DNA) >200,000IU/ml, corresponding to 5.3 log copies/l) is 70 to 90% [7]. Hepatitis B vaccination of newborns is an important method for preventing HBV infection worldwide [8, 9]. In contrast, MTCT has been identified as the cause of HBV vaccine failure [10, 11]. The world health organization has recommended tenofovir disoproxil fumarate (TDF) prophylaxis for pregnant women with high viral loads from the 28 ^th^ week of pregnancy until delivery in addition to the birth dose and routine vaccines for all neonates [12]. Therefore, either viral load or HBeAg determination is essential for the administration of tenofovir during pregnancy to prevent MTCT.

Hepatitis B virus is a member of the *Hepadnaviridae* family with a circular, partially double stranded DNA genome, size of 3.2 kb. Surface (S), Polymerase (P), Core and X proteins are encoded by four overlapping open reading frames (ORF) [13]. HBV replicates using reverse transcriptase, which has poor proof-reading capability. This results in a high level of genetic diversity, which has been classified into ten genotypes (A-J) [14], over forty-five sub genotypes [15] and nine serotypes [16]. Some HBV genotypes and sub-genotypes are found only in specific geographic areas, while others are found all over the world [17]. Genotypes A, and D are found worldwide while E is found mainly in Africa [18].

HBV genotypes and subtypes differ in terms of disease severity and treatment responsiveness [19]. Information on HBV genetic variation is important not only for epidemiological reasons, but also for understanding HBV transmission routes and developing efficient HBV prevention and treatment methods in the country. Drug resistance mutations, vaccine failure mutations, and viral escape mutations, on the other hand, may limit the effectiveness of current treatment and preventative efforts [20]. The situation in Ethiopia is worsened by significant public health concerns regarding the co-endemicity of the HIV and HBV, which usually affects the prognosis of infected individuals [21]. Before starting antiretroviral therapy (ART), the WHO recommends screening for hepatitis B surface antigen (HBsAg) in all HIV-positive individuals [22]. However, owing to a lack of suitable resources, such as diagnostic testing (HBV test kits, HBV viral load machine), and the high cost of HBV therapy, screening for HBV in HIV-positive individuals and during pregnancy is not frequently undertaken in Ethiopia. If HBV infected patients are co-infected with HIV and are not treated or exposed to antiretroviral medications, they are at risk of resistance associated mutations (RAMS) [23].

There have been a few molecular studies conducted among blood donors, and HIV co-infected patients in Ethiopia [21, 24–26]. However, little is known about the genetic diversity of HBV among pregnant Ethiopian women where the virus is endemic. Therefore, the goal of this study aimed to examine the viral load, and genetic diversity of HBV among pregnant women which has implications for programmatic and policy decisions.

## Materials and methods

### Study area, design and population

A cross-sectional study was conducted among pregnant women attending antenatal clinics at three tertiary hospitals located in major cities of the Amhara National Regional State in northwest Ethiopia. Gondar is home to the University of Gondar Comprehensive Specialized Hospital (UOGCSH). Debre Markos Referral Hospital (DMRH) is located in Debre Makos and Felege-Hiwot Comprehensive Specialized Hospital is located in Bahir Dar. The research was conducted between May 1, 2018 and September 30, 2019. The sample size was 1121 [31] but to increase the chance of positivity for molecular test, we have included 1846 pregnant women. Inclusion Criteria: Pregnant women who are HIV positive or HIV negative, able to give blood for screening of HBV and HCV were included in the study. Exclusion criteria: Pregnant women who were critically ill and unable to give blood were excluded from study.

### Ethics approval and consent to participate

Ethical approval for the study was obtained from Institutional review board (IRB) of University of Gondar (reference number VP/RCS/051756/2017). Written informed consent was obtained from each pregnant woman before enrolled in this study. The study participants’ anonymity was maintained throughout and participants were identified only by their code numbers. Confidentiality of information was maintained by locking the information using a computer password. Those participants with a positive result for HBsAg and HBV viral load were referred for better management.

### Clinical data, blood sample collection, and screening

After obtaining consent from the pregnant women, trained senior health workers collected sociodemographic data and five milliliters of venous blood. The WHO recommendation [27] was followed for the sample collection and processing. Whole blood was centrifuged at 3000 rpm for 10 minutes to separate serum. Separated serum samples were transferred to a cryotube and maintained in the refrigerator at -80°C until processing. Sera from 1846 pregnant women were screened for HBsAg using enzyme-linked Immuno sorbent assay (ELISA) according to the manufacturer’s instructions (Linear Chemicals, S.L.U., Spain). Serum samples were stored at -80°C at the University of Gondar`s Medical Microbiology Laboratory, and before being transported on dry ice to the Institute of Virology, Leipzig University, Germany.

### HBV DNA extraction, quantification, amplification and sequencing

The Magna Pure LC System (Roche Molecular Systems, Mannheim, Germany) was used to extract HBV DNA from 200 μL serum. As previously described [28], HBV DNA viral load was determined using Real time PCR with TaqMan probe detection format on Light cycler 2 system (Roche Molecular Systems, Mannheim, Germany) with forward 5’ACTCGTGGTGGACTTCTCTCA3’) and reverse primer (5’GAGGACAAACGGGCAACATACC3’) and TagMan-Probe (5’ 6FAM -CATCCTGCTGCTATGCCTCATCTTCT-BBQ 3’ with a lower detection limit of 50 IU/ml. For Sequencing, an approximately 911 bp long fragment corresponding to the overlapping HBV RT codon 60–298 and HBsAg codon 58–226 was amplified using Platinum-Taq-DNA polymerase (Thermofisher) with forward (5’ACTCGTGGTGGACTTCTCTCA3’) and reverse (5’GGGTTGCGTCAGCAAACAC3’) primers. With a final volume 50 μL, PCR reaction consisted of 28.1 μL of water (Braun), 5.0 μl of 10X Buffer, 1.5 mM of MgCl_2_, 1.0 μL of 10 mM dNTP’s, 10 pmol for each forward and reverse primer, 5U Taq polymerase, and 10 μL of template DNA. The PCR conditions were initial denaturation at 94°C for 2 minutes, followed by 45 cycles of denaturation at 94°C for 30 seconds, primer annealing at 58°C for 30 seconds, elongation at 72°C for 60 seconds and final elongation at 72°C for 5 minutes. Amplicons were sequenced bi-directionally using Big Dye Terminator v1.1 kits using an ABI PRISM 3500 DNA Analyzer (PE, Applied Biosystems, CA, USA). All sequences were submitted to Gen Bank (accession number: Op432532).

### HBV sequence analysis and genotyping

Nucleotide sequences were assembled manually and edited using the Geneious software version 11.0.4. HBV genotyping was done using NCBI genotypic tool and phylogenic tree analysis. After obtaining references from the HBV database and homologous sequences from the National Center for Biotechnology Information (NCBI) data base using a nucleotide BLAST (Basic Local Alignment Searching Tool). A phylogenetic tree was constructed using MEGA software version 7.0 and neighbor joining method [29]. HBV serotyping was determined using http://hvdr.bioinf.wits.ac.za/serotyper/. The surface gene (S) mutations that cross pond to diagnostic and immune escape mutants, as well as polymerase (rt) mutations that cross pond to HBV drug resistance, were determined using Geno2pheno analysis and HBV data base interpretation tools (accessed on January, 2021) and based on a previously published report [30].

## Results

### Sociodemographic characteristics

The study comprised 85 pregnant women who tested positive for HBsAg. More information on the serological test results and demographic characteristics was previously published [31]. Pregnant women were on average 25 years old, with a range of 17 to 45 years old. The majority 68(80%) of pregnant women was from urban dwellers. Most of the pregnant women, 79(92.9%) were married (Table 1).

**Table 1:** Sociodemographic and laboratory profile of HBsAg positive pregnant women.

Variables	n (%) or Average (range)
**Age in years (range)**	25(17–45)
**Residence**	
Urban	68(80%)
Rural	17(20%)
**Marital status**	
Married	79 (92.9)
Unmarried*	16(7.1)
Viral load in log IU/ml (range)	3.4 (2.6–7.6)
**HBV serotype**	
**adw2**, *n (%)*	39(84.8%)
**ayw2**, *n (%)*	5(10.9%)
**ayw3**, *n (%)*	2(4.3%)
**HBV genotype**	
Genotype A	39(84.8%)
Genotype D	7(15.2%)
**Sub genotypes**	
Sub genotype A1	84.8%
Sub genotype D2	4.3%
Sub genotype D4	2.2%
Sub genotype D10	8.7%

### Viral load, genotypes and serotypes

Of the 85 HBsAg positive pregnant women, 59 (69.4%) were positive for HBV DNA. The remaining 26 (30.6%) serum samples showed no detectable HBV DNA. The median viral load was 3.5 (2.6–7.6) log IU/ml among the 59 detected viral loads. The majority of samples 39 (66%) had viral load below 4 log or 10^4^ IU/ml. Fifty-nine cases were amplified, with 46 of them being sequenced successfully. Based on the phylogenetic analysis, 39 (84.7%) of the isolates classified under genotype A whereas 7(15.3%) were belonged to genotype D (S1 Fig). The subtype analysis showed that all genotype A classified into A1 (84.8) sub-genotype unlike genotype D had three sub-genotypes 2 (4.3%) D2, 1 (2.2%) D4, and 4 (8.7) D10 (Table 1).

The phylogenetic analysis also showed that majority of genotype A strains phylogenetically related to strains from Haiti, Philippines, India, Japan, Kenya, South Africa, Brazil, Uruguay, Italy and Germany. Interestingly, one strain (99000271BD704 ETH) seems to be an old strain that preceded other strains found in different countries of the world.

### Immune escape HBsAg and drug resistance gene mutations

Many mutations were detected in the Major Hydrophilic Region (MHR) of the surface genes, and reverse transcriptase of polymerase gene region. Nineteen out of forty-six (41.3%) cases were one or more mutations in the MHR. Of which, 6 (13.1%) of the cases were mutations (P127T, Q129R, N131T, Y134S, F134Y, F134L, T140I, and T143S) with in “a “determinant region (aa 124–147) of MHR. The remaining mutation was detected (aa 99–200) out of the determinant region (S1 Table). Five cases (10.9%) were mutations that have a potential impact on detection of HBsAg: sM103I, sP120T, sP120S, sQ129R/H, sF134L, sE164G and while vaccine or immune escape associated mutants were detected in 6 (13.0%) cases: sP120S, sP120T, sQ129H/R, sN/T131S/I, sY134F, sY134S, sT140I, sT143M. Interestingly, we also isolated drug resistance mutations in 6(13.0%) cases in reverse transcriptase region of polymerase gene. In addition, 2 (3.4%) of the cases were antiviral drug-associated potential vaccine escape HBV mutations (ADAPVEMS) in HIV infected and non-infected patients. The mutations detected were: rtM204V/sI195M, rtM204V/sT118M, rtM204V/sQ129H/R, rtM204V/ sM103T, rt V173L/L180M/M204V + sE164D/sI195M.

## Discussion

The viral load of pregnant women in this study ranged from 2.6–7.6 log IU/ml. This implies that some pregnant women had a high viral load that potentially could have a high chance of MTCT of HBV. Growing evidence showed that in a pregnant woman with HBV viral load greater or equal to 5.3 log IU/ml or 200,000 IU/ml the chance of MTCT is high [7, 12]. Notably, serum HBV viral load level has been identified as a single independent risk factor for MTCT [32]. Viral factors like the presence of high viral load or HBeAg in serum of pregnant women are responsible for perinatal transmission of HBV [33].

Without any intervention, 70–90% of infants born to mothers positive for both HBsAg and HBeAg will acquire the infection perinatally in endemic countries [7]. HBV is a major public health burden in developing countries and decreasing the MTCT is a key strategy to reduce the prevalence and burden of the infection. The growing evidence showed that administration of HBIG and HBV vaccine within 24 hours of birth is effective to prevent more than 85% of MTCT [34]. To prevent transmission of MTCT, WHO recently recommended tenofovir to be given for pregnant women in the third trimester [12]. However, in resource limited countries like Ethiopia, tenofovir and HBIG are not feasible due to high cost, availability and cold-chain issues. Therefore, the authors recommend that at least HBV birth dose vaccine shall be started and scaled up in all health institutions in the country to reduce MTCT.

Current study revealed that genotype A (84.8%) was predominant which is similar with prior studies done among blood donors and HIV infected patients in Ethiopia [21, 24, 25, 35]. This finding is also consistent with neighboring countries Kenya, Uganda and Tanzania [36–39]. On the contrary, genotype D is predominantly detected in occult hepatitis HIV-infected patients and pregnant women in Ethiopia [40, 41] and other neighboring countries Eritrea and Sudan [42, 43]. Genotype E was not found in the current study. However, genotype E has been documented in Ethiopian investigations [21, 35, 41]. Similarly genotype C was not detected in current study but it was reported in another study in Ethiopia [41]. The variation in genotypes in Ethiopia and other countries could be attributed to a variety of factors an international travel, different risk factors, migration, war and tourism [44]. To note that the genotype distribution of Africa, genotype A is mainly found in South Africa, East and Central Africa, while Genotype D mainly in North Africa and Genotype E in Western Africa [45].

All genotype A isolates in our study belonged to sub-genotype A1. This is supported by research conducted in Ethiopia and Kenya [24, 25, 37]. Interestingly, the majority of the Ethiopian A1 strains in this study clustered with Asian-American clade. They were closely related to Haiti, Philippines, India, Brazil, Japan, Kenya, South Africa, United States of America, Uruguay and Bangladesh. This finding might strengthen the hypothesis that the distribution of A1 strain from East Africa to other countries is likely through slave trade and migration [46]. Genotype A is highly heterogeneous and has been classified in to seven sub-genotypes [44]. Sub-genotypes A1 were originally identified in Southern Africa and circulate in Eastern Africa [37]. Evidence suggests that genotype A is associated with hepatocellular carcinoma and the most common genotype in sub-Saharan Africa [47]. Ethiopia belongs to sub-Saharan African countries, the mortality due to HCC is the highest in the world, where genotype A is predominant and aflatoxin is also common [48].

Growing shred of evidence showed that, genotype D is the most common genotype in North Africa, Middle East and the Mediterranean Sea, India, and Sudan [43, 49]. In the current study genotype D is common in Ethiopia next to genotype A. This finding is similar to studies done previously [21, 24–26]. Our study revealed that genotype D is sub divided into sub genotypes D2, D4 and D10. Surprisingly, a recently identified new sub-genotypes D10 circulating in Ethiopia [50] was also detected in this study. A study in Ethiopia found that D1, D2, D4, D6, D7 and D10 among blood donors [50] and another recent study in Ethiopia reported sub genotype D1, D2, D4, D6 among blood donors, HIV-infected patients and chronic liver disease patients [35]. This implies that more diversified sub-genotypes of D emerged in Ethiopia possibly due to mutation, and an old genotype exist in the country. Interestingly, this study also characterized the serotype or subtypes of HBV among pregnant women. Subtype adw2 was the predominant (84.8%) and isolated in all genotype A. Likewise subtype ayw2 and ayw3, 4 (10.9%) and 2(4.3%) belonged to D genotypes; respectively. This finding is consistent with other studies [24, 25].

Moreover, knowing mutations of HBsAg and RT region of HBV is becoming more important in the recent times to predict escape of detection, vaccine escape and drug resistance. Documented pieces of evidence showed that mutants within the MHR region of surface gene is associated with HBsAg detection failure, vaccine and Hepatitis B immunoglobulin (HBIG) escape [24, 51, 52]. Mutations of HBsAg causing immune escape can also be the result of changes in the RT due to antiviral therapy because of the overlapping reading frames [53]. Interestingly, in the present study, 19/46 (41.3%) of amino acid substitution were identified in MHR of HBV S gene and nearly half of the mutation were observed in “a” determinant region. Similarly, studies reported mutations of MHR of surface gene among blood donors, HIV-infected and chronic liver patients in Ethiopia [24–26, 54]. This implies that mutation in MHR and “a” determinant region may pose a challenge on some commercial serological diagnosis of HBV and vaccine escape on the community.

Furthermore, mutations related to antiviral therapy were detected in 6/46 (13.0%), triple mutations rtV173L, rtL180M, and rtM204V resistant to lamivudine, double mutation; rtL180M and rtM204V, resistant to entecavir and M204V resistant to telbivudine in one pregnant woman whom was on ART drugs. Similarly, studies reported drug resistance mutation among drug naïve and in HIV-infected patients in Ethiopia and Nigeria [21, 24, 26, 54]. Surprisingly, we have isolated R153W and N248H mutations in this study similarly a study done in Nigeria [54]. These R153W and N248H drug resistance mutations were not detected in previous studies in Ethiopia. Furthermore, a systematic and meta-analysis reported drug resistance mutation and vaccine escape mutation in Africa. A meta-analysis on genetic variability of RT/ HBsAg overlapping region of hepatitis B virus (HBV) isolates of Bangladesh [56]. They stressed that there is an urgent need for improved diagnosis, vaccination and treatment [55, 56].

This implies that mutations resulted in vaccine, diagnosis escape and drug resistances were increasing from time to time. HBV infected patients remain untreated in Ethiopia or exposed for antiretroviral drugs if co-infected with HIV, these situations expose them at risk of resistance associated mutations. Taken together, this is imperative that screening for HBV among HIV-infected pregnant women is essential to tackle drug resistance of HBV. As limitation of this study, HBeAg and occult HBV status were not known in these pregnant women.

## Conclusion

This study revealed that genotype A was isolated predominantly among pregnant women. Mutations were detected on surface gene and reverse transcriptase gene that may lead to failure of serology detection, immune therapy escape and treatment of HBV. Determining viral load, genotyping and assessing mutations of surface and polymerase genes of HBV and early treatment of pregnant mothers, giving birth dose vaccine for infants are important for prevention of MTCT. Therefore, large-scale longitudinal studies are needed on HBV gene mutation, vertical transmission, and treatment and vaccine effectiveness to curve and eliminate the disease.

## Supporting information

S1 Fig Rooted phylogenetic tree constructed by neighbor-joining method based on partial S/P genes sequences of HBVstrain. Ethiopian strains indicated by diamond shaped symbol and the reference sequences used as a comparison represented by black circle, accession number followed by corresponding genotypes with capital letter. Bootstrap resampling was carried out 1000 times and only bootstrap values above 70% indicated on the respective nodes.
(TIFF)

S1 Table Distribution of HBV mutations detected on the surface and polymerase genes among pregnant women from May1, 2018 to September 30, 2019. (DOCX)
