# Supplementary material for: Molecular diversity of hepatitis B virus among pregnant women in Amhara National Regional State, Ethiopia
Source: PLoS One. 2022 Nov 15;17(11):e0276687. doi: 10.1371/journal.pone.0276687 (PMC9665361; doi:10.1371/journal.pone.0276687)
Supplement: S1 Table — (DOCX) [file pone.0276687.s002.docx]

Table 2. Distribution of HBV mutations detected on the surface and polymerase genes among pregnant women from May1, 2018 to September 30, 2019.

| HBV S/P gene region | | Mutation | Sample/sequence identification |
| --- | --- | --- | --- |
| MHR (aa 99-169) | `a` determinant region (aa 124-147) | sP127T | 9900271 |
|  |  | sQ129R | 9900369 |
|  |  | sN131T | 9900271, 990272 |
|  |  | sY134S | 990044 |
|  |  | sF134Y | 990271 |
|  |  | sF134L | 9900367 |
|  |  | sT140I | 9900301 |
|  |  | sS143T | 9900367 |
|  | Outside `a' determinant region (aa. 99±123 & 148±169) | sY100C | 9900301 |
|  |  | sM103T | 9900369 |
|  |  | sM103I | 9900377 |
|  |  | sI110L | 9900294 |
|  |  | sT118A | 990044, 9900280 |
|  |  | sT118M | 9900369 |
|  |  | sP120T | 990044 |
|  |  | sK122R | 9900271 |
|  |  | sY161F | 9900271,9900272 |
|  |  | sE164D | 9900369 |
| Table 5 continued | | | |
| Downstream of the MHR (aa170-221) | | sS174NS | 9900301 |
|  |  | sV184A | 9900384,9900463 |
|  |  | sP188L | 9900463 |
|  |  | sS193L | 900463 |
|  |  | sA194V | 9900531 |
|  |  | sI195M | 9900369 |
|  |  | sM198I | 9900463 |
|  |  | sY200F | 9900286, 9900296 |
|  |  | sS204N | 9900273 |
|  |  | sY206H | 9900298 |
|  |  | sS207N | 9900128,9900272,  9900273,9900182, 9900164, 9900369 |
|  |  | sL216* | 9900294 |
| RT region/drug resistance mutation | | rtV173L | 9900369 |
|  |  | rtL180M | 9900369 |
|  |  | rtM204V | 9900369 |
|  |  | rtR153W | 990045,90080,9900291 |
|  |  | rtN248H | 90044,990045, 990080,9900298 |
